# Death from Alveolar Hemorrhage

**Published:** 1872-04

**Authors:** 


*Death from Alveolar Hemorrhage.*—In New York city, on the 26th of February last, a German named Charles Nollman, had five teeth extracted. On leaving the dental office the gums were bleeding profusely, and all attempts to arrest the hemorrhage were in vain. On Wednesday, Thursday, and Friday, the bleeding continued, and although every effort was made to save the unfortunate man's life by Dr. Chabert, a prominent physician of Hoboken, who applied styptics, and did all that medical science could suggest. he became weaker and weaker, and at last expired from inanition. A few hours before his death, two friends of the patient allowed themselves to be bled, each to the extent of eight ounces, for the purpose of seeing if Nollman's life could be saved by the transfusion of blood; but the unfortunate patient was too far gone, being then in a dying condition. A hemorrhagic diathesis was clearly present in this case.

